# A Treatise on Fractures

**Published:** 1859-08

**Authors:** 


ART. VI.—*A Treatise on Fractures.* By J. F. MALGAINE, *Chirurgien de l'Hôpital Saint Louis, Chevalier de la Légion d'Honneur et du Mérite Militaire de Pologne, Membre de l'Académie Royale de Medicine.* With one hundred and six illustrations. Translated from the French, with Notes and Additions, by JOHN H. PACKARD, M.D. Philadelphia: J. B. Lippincott & Co. 1859.

The subjects of fractures and dislocations are, perhaps, themost important of all the departments of surgery, especially as regards anxiety to the medical attendant during the processes of treatment, and the importance of the result of such cases to his reputation, and sometimes to his pocket. Almost every suit for malpractice is a case either of deformity after fracture, or of an old dislocation; and the necessity has long been felt of a work which should be an authority for the practitioner, and serve to instruct our intelligent juries as to what we may expect as the result of the various kinds of fractures, and what is good and approved treatment. The work of Malgaine, who is perhaps the highest authority on this subject in the world, the translation of which has been so ably performed by Dr. Packard, will now supply the deficiency to which we have alluded; and though there is a work in press on fractures and dislocations, by Dr. Hamilton, of Buffalo, who is more closely identified with that subject than any man in America, the profession should no less appreciate the valuable service which it has received in the translation of Malgaine.

All that we will be able to do in our notice of the work before us, will be to give a general outline of its arrangement, and our estimate of its great value. Its plan differs somewhat from that generally adopted by English and American authors. First, we have the consideration of fractures in general, commencing with etiology. The general predisposing causes which influence these accidents, such as age, sex, the seasons, anatomical predispositions, cachexiæ, scorbutic, gouty, cancerous, etc., with the various determining causes, are fully and ably dwelt upon; and the author then proceeds to the consideration of the "varieties of fractures." Here we have an arrangement not found in English or American text-books, more simple than that which is commonly adopted, and we think, on the whole, more desirable. We have fractures divided under the heads of "incomplete; complete, but simple; multiple; and complicated." Under the head of incomplete fractures, we have fissures, splintered fractures, and perforations; of complete, simple fractures, we have transverse, serrated, and oblique fractures, as separations of epipyses; multiple fractures include, fractures with splinters, fractures of one bone in several places, fractures by crushing, and fracture of several bones at once; finally, complicated fractures comprise those with which we have connected a local lesion or any disturbance of the economy. This embraces eighty pages, and the various points which we have enumerated, are fully discussed, and illustrated from the store of cases which the long experience of M. Malgaine has placed at his disposal. The translator has also interpolated some cases which have been reported in this country, which, of course, have not come under the observation of the author. American literature has been so little known in France, especially in the practical department, that many of our interesting cases and valuable contributions to science are entirely lost to the French authors. Here, indeed, translator's notes are almost necessary to make a work complete for American readers, though we have often taken a decided ground against copious additions to republications of foreign works, and are gratified to see that a sentiment which is quite general is now beginning to meet with some attention from our large publishers, who, in some instances, have actually stricken out editorial notes which appeared in former editions ; for example, Erichsen's Surgery, edited by Dr. Brinton, of Philadelphia, a late edition of which has appeared without the editor's notes.

What we have just sketched is embraced in articles first and second of chapter first. Article third is devoted to the general semiology of fractures, comprising the cracking at the occurrence of the injury, pain, loss of power, contusion and ecchymosis, preternatural mobility, deformity, and crepitation. The subject of deformity, as well as that of crepitation, is minutely considered. The author is not disposed to attribute to muscular action the great power which is generally ascribed to it by anatomists in producing deformity after fractures; and he thinks that the direction, extent, etc., of the force which causes the fracture, the resistance of the periosteum, the impaction of the serrations, and many accidental influences, have much to do with the production of deformity; and, as resulting from from this opinion, he concludes : 1. "that every displacement, except overlapping, can be reduced and obviated, if only the fragments afford sufficient hold. 2. That overlapping is the most stubborn of them all. It is a disagreeable truth, generally too much kept out of sight by the classical author, that overlapping is so stubborn as to baffle the effects of art to overcome it, in the great majority of cases." That this latter clause is eminently true, has been now taught to American surgeons by Prof. Hamilton, who has clearly shown in his able and voluminous reports to the American Medical Association on "Deformities after Fractures," that in the great proportion of oblique fractures, and in nearly all oblique fractures of the thigh in the adult, it is impossible to prevent overlapping and consequent shortening of the limb. It is also a well-established fact, that fractures of the clavicle are most commonly followed by deformity. So well known is it, that some authorities dispense with all retentive apparatus, and are satisfied merely to keep the patient as quiet as possible. What we have seen of these accidents, leads us to the opinion that the axillary pad, and the various complicated apparatus for retaining the fractured ends of the bone in place, are sometimes productive of more harm than good, while simple measures are quite as effectual.

Articles fourth, fifth, and sixth, are respectively devoted to the course and terminations, diagnosis, prognosis, and treatment of fractures. In each of these divisions we have an elaborate consideration of the subject of which it treats. This finishes the first chapter, which contains two hundred and eighty-seven pages, devoted entirely to general considerations, nearly half of the entire volume. This may seem a large portion to devote to generalities, and will perhaps intimidate some of the students of this subject; but when we consider the importance of the subject of fractures, the imperfect manner in which they are considered by systematic writers on surgery, and especially the necessity of adapting a plain common sense with a good general idea of anatomical difficulties to be surmounted, and the indications to fulfill in fractures generally, to the treatment of individual cases, the space devoted to generalities does not seem too considerable.

The remainder of the work, nearly four hundred pages, is devoted to special fractures. We had not the intention of making a critical analysis of this treatise, desiring only to give our readers an idea of its scope and arrangement. Suffice it to say, as regards the remainder, that all the individual fractures are treated of in respect to frequency, diagnosis, prognosis, and treatment. We cannot discuss the views of the author without extending our notice beyond the limits to which we are confined. We can only say that he is one of the highest, if not the highest, living authority upon this subject; and the reader, in consulting this work, will find more practical information than in any other work in our language.

We cannot close without expressing our appreciation of the able manner in which the laborious work of translation has been performed by Dr. Packard. We have not compared it with the original, but are convinced from its pervading clearness of expression, that we have a faithful translation. This point should always be kept in view by translators, who are sometimes led to improve upon the author's mode of expression, a little more than is admissible in avoiding peculiar idiomatic expressions. Editorial notes, also, are not numerous, and when they occur, serve to illustrate the author's statements, by cases to which he could not have had access. A criticism which we felt inclined to make after an examination of the work, is in the disposition of the illustrations. We think they would have been better placed, interspersed in the text, than collected at the end of the volume. This, however, is a minor consideration. The engravings have been reduced from a folio atlas which accompanies the French edition and comprises engravings of a number of rare specimens in the possession of M. Malgaigne, or to be found in the museums. The mechanical execution of the engravings, as well as that of the body of the work, is excellent, and does credit to the enterprising publishers, Messrs. J. B. Lippincott & Co., of Philadelphia.

